# Favipiravir Suppresses Zika Virus (ZIKV) through Activity as a Mutagen

**DOI:** 10.3390/microorganisms11051342

**Published:** 2023-05-19

**Authors:** Evelyn J. Franco, Eleonora Cella, Xun Tao, Kaley C. Hanrahan, Taj Azarian, Ashley N. Brown

**Affiliations:** 1 Institute for Therapeutic Innovation, Department of Medicine, College of Medicine, University of Florida, Orlando, FL 32827, USA; e.franco@ufl.edu (E.J.F.); kaley.hanrahan@medicine.ufl.edu (K.C.H.); 2 Department of Pharmaceutics, College of Pharmacy, University of Florida, Orlando, FL 32827, USA; tealingsxun@gmail.com; 3 Burnett School of Biomedical Sciences, University of Central Florida, Orlando, FL 32827, USA; eleonora.cella@ucf.edu (E.C.); taj.azarian@ucf.edu (T.A.)

**Keywords:** favipiravir, Zika virus, mechanism of action, mutagen, defective viral particle

## Abstract

In a companion paper, we demonstrated that the nucleoside analogue favipiravir (FAV) suppressed Zika virus (ZIKV) replication in three human-derived cell lines—HeLa, SK-N-MC, and HUH-7. Our results revealed that FAV’s effect was most pronounced in HeLa cells. In this work, we aimed to explain variation in FAV activity, investigating its mechanism of action and characterizing host cell factors relevant to tissue-specific differences in drug effect. Using viral genome sequencing, we show that FAV therapy was associated with an increase in the number of mutations and promoted the production of defective viral particles in all three cell lines. Our findings demonstrate that defective viral particles made up a larger portion of the viral population released from HeLa cells both at increasing FAV concentrations and at increasing exposure times. Taken together, our companion papers show that FAV acts via lethal mutagenesis against ZIKV and highlight the host cell’s influence on the activation and antiviral activity of nucleoside analogues. Furthermore, the information gleaned from these companion papers can be applied to gain a more comprehensive understanding of the activity of nucleoside analogues and the impact of host cell factors against other viral infections for which we currently have no approved antiviral therapies.

## 1. Introduction

Zika virus (ZIKV) is a positive-sense single-stranded RNA virus in the family Flaviviridae that is primarily transmitted by Aedes mosquitoes [1,2]. ZIKV’s initial discovery in 1947 was followed by a long stretch of relative obscurity since documented cases of infection were infrequent until the first reported outbreak in 2007 [3,4,5]. The next decade saw Zika virus rise to public consciousness following outbreaks that introduced the virus to the Pacific in 2013/2014 and the Americas in 2015/2016 [2,3].

Infection is estimated to produce symptoms in approximately 20% of patients [6,7]. Based on accounts from initial case reports, it was believed infection with ZIKV caused a mild febrile illness [8] characterized by symptoms including fever, rash, headache, and joint and muscle pain [5,8]. However, concerns over the sequelae of infection increased during larger outbreaks due to evidence indicating that ZIKV could be transmitted vertically from mother to fetus [5,9,10] and that fetal infection was associated with devastating consequences including a higher risk for birth defects, microcephaly, and developmental delay [9,11,12]. Throughout the course of these larger outbreaks, it also became apparent that ZIKV infection was linked to a higher risk for neurologic complications in adults since the incidence of Guillain–Barré syndrome increased among infected patients [5].

Although the infection has the potential to substantially impact patient quality of life, antiviral therapies or vaccines to combat the disease have not yet been approved [5]. Treatment recommendations remain non-specific and are centered on supportive care measures that may increase patient comfort but do not speed recovery or target viral replication [13,14].

Work conducted by our lab as well as others has shown that among available antivirals, the nucleoside analogue favipiravir (FAV) is one of the most promising antiviral candidates against (re-)emerging viral infections since it has exhibited broad-spectrum activity against several families of RNA viruses [15,16,17,18,19,20]. In spite of FAV’s demonstrated effect against a range of viruses, our previous work has revealed that rather than eliciting uniform activity when comparing results from studies conducted in cells derived from different tissues and species, susceptibility to FAV’s effect is highly influenced by host cell line [18]. This finding prompted us to evaluate FAV’s antiviral potential against ZIKV in HeLa, SK-N-MC, and HUH-7 cells, three cell lines derived from human tissue [21]. The results of our companion study showed that FAV successfully hindered ZIKV replication in all three cell lines, but predictably, the amount of suppression achieved in each cell line was variable. Viral inhibition was achieved in a clear dose-dependent manner in all three cell lines; however, results in HeLa cells were distinct in that we also observed enhanced antiviral effect over time, a phenomenon that was absent in the results from studies carried out in SK-N-MC and HUH-7 cells. Further investigation showed that drug exposure caused a considerable loss of viral infectivity in HeLa cells, while only slight declines in infectivity were achieved in SK-N-MC and HUH-7 cells, suggesting that FAV-induced loss of viral infectivity contributes to FAV’s potent anti-ZIKV activity in HeLa cells.

To our knowledge, FAV’s antiviral mechanism of action against ZIKV has not been characterized. Although it is known that FAV activity is mediated through the interaction of the active triphosphate metabolite, FAV-RTP, with the catalytic site of viral polymerases [22,23,24], FAV’s antiviral mechanism of action remains a topic of debate. Work conducted by others has suggested that incorporation of FAV-RTP into nascent strands of viral RNA may inhibit viral replication through multiple mechanisms as reports of FAV activity as either an inducer of lethal mutagenesis [25,26,27,28,29] or a chain terminator [30,31] have been documented. Based on FAV’s observed effects on viral infectivity, we hypothesized that FAV may be acting as a mutagen and that the accumulation of mutations in viral genomes over time leads to an increase in the production of defective viral particles, non-infectious viruses with the ability to suppress replication of infectious virus through competition for receptors and cellular and viral proteins [32,33,34]. In these studies, we aim to identify the mechanism of action relevant to FAV’s activity against ZIKV as well as characterize the host cell factors contributing to tissue-specific differences in susceptibility to FAV.

## 2. Materials and Methods

### 2.1. Cell Lines

The HeLa cell line was obtained from the American Type Culture Collection (ATCC CCL-2; Manassas, VA, USA) and was maintained in minimum essential medium (MEM; Corning Cellgro; Manassas, VA, USA) supplemented with 10% fetal bovine serum (FBS; Sigma Aldrich; St. Louis, MO, USA) and 1% penicillin–streptomycin solution (Hyclone; Logan, UT, USA). SK-N-MC cells were obtained from ATCC (HTB-10) and maintained in MEM supplemented with 10% FBS (Sigma Aldrich; St. Louis, MO, USA), 1% penicillin–streptomycin solution (Hyclone; Logan, UT, USA), 1% sodium pyruvate (Hyclone; Logan, UT, USA), and 1% non-essential amino acid solution (Hyclone; Logan, UT, USA). HUH-7 cells were obtained from the JCRB Cell Bank (JCRB0403) and were maintained in Dulbecco’s Modified Eagle’s Medium (DMEM; Corning Cellgro; Manassas, VA, USA) supplemented with 5% FBS (Sigma Aldrich; St. Louis, MO, USA) and 1% penicillin–streptomycin solution (Hyclone; Logan, UT, USA). Finally, Vero cells (ATCC CCL-81) were maintained in MEM (Corning Cellgro; Manassas, VA, USA) supplemented with 5% FBS (Sigma Aldrich; St. Louis, MO, USA) and 1% penicillin–streptomycin solution (Hyclone; Logan, UT, USA). Cells were maintained at 37 °C and 5% CO_2_. Cells were split twice weekly to maintain subconfluency.

### 2.2. Virus

The 2015 human ZIKV Puerto Rican strain, PRVABC59, was obtained from Biodefence and Emerging Infections Research Resources Repository (BEI Resources; Manassas, VA, USA). Viral stocks were propagated on Vero cells (ATCC CCL-81) as described previously [35].

### 2.3. Antivirals

FAV was purchased from MedKoo Biosciences Inc. (Morrisville, NC, USA) and stored according to manufacturer specifications. FAV stocks of 100 mM were prepared in 100% dimethyl sulfoxide (DMSO).

### 2.4. Antiviral Evaluations

HeLa, SK-N-MC, and HUH-7 cells were seeded into six-well plates and incubated at 37 °C and 5% CO_2_ overnight. Comparable viral replication kinetics were maintained between tissue types by infecting HeLa and SK-N-MC cells at a multiplicity of infection (MOI) of 1 while HUH-7 cells were infected at an MOI of 0.1. The virus was allowed to adsorb onto cells for one hour, and then the viral inoculum was removed and monolayers were washed twice with warm phosphate-buffered saline (PBS) to remove unbound virus. Then, 3 mL of FAV-containing medium was added to wells, and plates were incubated at 37 °C and 5% CO_2_. Viral supernatants were sampled daily, clarified by high-speed centrifugation, and stored at −80 °C until the end of the study. Assays were conducted in triplicate in two independent assays. Due to day-to-day variability in time to peak viral titers in HUH-7 cells, one experiment was carried out for a total of four days, while the second was ended after three days of infection; however, it must be noted that peak infectious titers of approximately 8 log_10_ PFU/mL were achieved in both assays.

### 2.5. Quantitative Real-Time Reverse Transcription PCR

Viral RNA was isolated from supernatant samples using the QIAamp Viral RNA mini kit (Qiagen; Germantown, MD, USA), and quantitative real-time reverse transcription PCR was performed on samples. Viral RNA was amplified using TaqMan Fast Virus 1 Step Master Mix (Applied Biosystems; Foster City, CA, USA) and primer–probe sets targeting either the 5′-untranslated region (UTR) or the 3′-UTR of the ZIKV genome (Integrated DNA Technologies; Coralville, IA, USA) [36]. Sequences of the 5′-UTR primer–probe set are as follows: primers 5′-CAGACTGCGACAGTTCGAG-3′ (forward, 1000 nM) and 5′-AGAAACTCTCGYTTCCAAATCC-3′ (reverse, 1000 nM); probe 5′-/56-FAM/CCTGTTGAT/ZEN/ACTGTTGYTAGCTYTCGCTTC/3IABkFQ/-3′ (250 nM). Sequences of the 3′-UTR primer–probe set are as follows: primers 5′-AAGCCATGCTGCCTGTGAG-3′ (forward, 1000 nM) and 5′-CAGATTRAAGGGTGGGGAAG-3′ (reverse, 1000 nM); probe 5′-/56-FAM-TTGGARGCG/ZEN/CAGGATGGGAAAAGAAGGT/lABkFQ/-3′ (250 nM). Thermal cycling conditions were 50 °C for 5 min, 95 °C for 20 s, followed by 40 cycles of 95 °C for 3 s and 60 °C for 30 s. Reactions were analyzed using the ViiA7 Real Time PCR System (Applied Biosystems; Foster City, CA, USA). Viral burden was predicted relative to a standard curve generated from serial 1:3 dilutions of ZIKV stock ranging from 6.97 to 1 × 10^8^ plaque-forming units per milliliter (PFU/mL).

### 2.6. Viral Genome Sequencing

RNA extracts were also used for viral genome sequencing. After RNA extraction, we performed cDNA synthesis and amplicon generation using a previously described ZIKV sequencing primer scheme [37]. Amplicons were quantified using a Qubit dsDNA HS Assay Kit (Thermo Fisher Scientific Inc., Waltham, MA, USA). Sequencing libraries were constructed with the Illumina Nextera Flex kit (Illumina Inc., San Diego, CA, USA) and sequenced on the Illumina MiSeq System (Illumina Inc., San Diego, CA, USA) to generate 2 × 250 nt reads to span the 400 nt amplicons. After sequencing, per-sample idealized coverage was calculated and quality was assessed using FastQC. Raw reads were quality filtered with Trimmomatic v.0.39 using the following settings: SLIDINGWINDOW:10:20 MINLEN:31 TRAILING:20 [38].

For each tissue, sequencing reads from the no-treatment control arm on day 1 were mapped to the ZIKV strain PRVABC59 reference genome (Genbank *MH158237*) using minimap2 [39]. Consensus genomes were then generated using the iVAR pipeline v1.3.1 [40]. These consensus sequences were used as the reference for mapping reads from the subsequent time points and drug concentrations (i.e., mutations were identified relative to the viral population from the no-treatment control arm on day 1 from each tissue type, thus allowing for identification of mutations that appeared in FAV-treated arms but were absent from the no-treatment control population). Each identified mutation was annotated, and a mutational pattern analysis for each time point/drug concentration was performed to quantify the number of transition and transversion mutations. R [41] with ggplot [42] was used for data management and visualization of mutational patterns. Transition/transversion (Ts/Tv) statistics and the overall number of mutations for the viral population were determined for each time point and drug concentration using VCFtools v0.1.13 [43]. For statistical comparison of each time point, we calculated the mean ts/tv ratio and number of mutations using the values for each drug concentration. For the mean values, the 95% confidence interval was calculated using the t-score due to the small sample size. The values were then compared to the 0 drug concentration for the same time point to determine significance.

We further investigated the presence of defective viral particles using DI-tector v0.6 [44]. DI-tector uses sequencing data to detect insertions/deletions that result in defective interfering (DI) genomes and copy-back (cb)/“hairpin” or snap-back (sb) DI genomes. The prevalence of defective particles was determined by calculating the ratio between the number of DI genomes and the amount of infectious virus in each sample.

### 2.7. Defective Particle Competition Assays

Six-well plates were seeded with HeLa, SK-N-MC, and HUH-7 cells at a density of 1 × 10^6^ cells/well and incubated at 37 °C and 5% CO_2_ overnight. The following day, cells were infected with ZIKV at an MOI of 1 for HeLa and SK-N-MC cells and 0.1 for HUH-7 cells. The virus was propagated either in the absence of FAV or in the presence of FAV at concentrations of 250, 500, or 1000 μM. Viral supernatant samples were collected at various time points and stored at −80 °C until the end of each study. The amount of virus in each sample was predicted by qRT-PCR. Briefly, viral RNA was isolated from supernatant samples using the QIAamp Viral RNA mini kit (Qiagen; Germantown, MD, USA) as per manufacturer recommendations. Then, viral RNA was amplified using TaqMan Fast Virus 1 Step Master Mix (Applied Biosystems; Foster City, CA, USA) and a primer–probe set targeting the 5′-untranslated region (UTR) of the ZIKV genome (Integrated DNA Technologies, Coralville, IA, USA): primers 5′-CAGACTGCGACAGTTCGAG-3′ (forward, 1000 nM) and 5′-AGAAACTCTCGYTTCCAAATCC-3′ (reverse, 1000 nM); probe 5′-/56-FAM/CCTGTTGAT/ZEN/ACTGTTGYTAGCTYTCGCTTC/3IABkFQ/-3′ (250 nM). Thermal cycling conditions were 50 °C for 5 min, 95 °C for 20 s, followed by 40 cycles of 95 °C for 3 s and 60 °C for 30 s. Reactions were analyzed using the ViiA7 Real Time PCR System (Applied Biosystems; Foster City, CA, USA). Viral burden was predicted relative to the amount of RNA present in each sample using a standard curve generated from serial 3-fold dilutions of ZIKV stock ranging from 2.32 to 1 × 10^8^ plaque-forming units per milliliter (PFU/mL).

Following quantification of virus levels by qRT-PCR, predicted viral burden data were used to dilute no-treatment control (NTC) and FAV-exposed (Tx) viral samples such that titers in the NTC and Tx virus samples matched. Of note, because numerous dilutions were required to match titers between NTC and Tx virus samples, residual levels of FAV in each sample were well below effective concentrations. To test for the presence of defective particles and characterize their interfering activity on viral replication, NTC virus was combined with increasing ratios of Tx virus. Then, 100 μL of each viral mixture was inoculated onto confluent monolayers of Vero cells and incubated for 1 h at 37 °C and 5% CO_2_. Following the 1 h incubation period, 3 mL of a primary MEM agar overlay containing MEM, 5% FBS, and 0.6% agar was added to each well. Plates were incubated for three days after infection, and then 3 mL of a secondary MEM agar overlay consisting of MEM, 1% FBS, 1% agar, 200 μg/mL DEAE-dextran, and 0.008% neutral red was added to each well. Plaques were counted 24 h after the addition of the secondary overlay. Infectious viral burden is reported as plaque-forming units per milliliter (PFU/mL). Infectious viral burden in viral mixtures was normalized against the viral burden of the sample containing 100% NTC virus at each time point.

## 3. Results

### 3.1. FAV Exhibits Mutagenic Activity against ZIKV

Cell-free ZIKV RNA isolated from viral particles released from infected HeLa, SK-N-MC, and HUH-7 cells was sequenced to address the hypothesis that FAV inhibits viral replication through activity as a mutagen. Analysis of the number of mutations in the virus released from infected HeLa cells revealed that ZIKV genomes were stable, as the number of mutations acquired throughout multiple rounds of replication was low, ranging from 21 on day 1 to 25 on day 5 in the absence of drug pressure (Table 1). Comparison of the mutational burden between the no-drug control and FAV-exposed arms showed that the number of mutations was similar on day 1; however, we detected a significant increase in the viral mutational burden propagated in the presence of FAV after 3 and 5 days (Table 1). Results in SK-N-MC and HUH-7 cells demonstrated that in the absence of the drug, ZIKV accumulated more mutations in these cell lines, as the mutational burden nearly doubled from day 1 to day 5 in SK-N-MC cells and increased by approximately 3-fold from day 1 to day 4 in HUH-7 cells (Table 1). The addition of FAV further increased the number of mutations detected in the virus released from these tissues. For example, FAV exposure led to a statistically significant increase in the mean mutational burden of viruses harvested from SK-N-MC cells, resulting in 1.5-fold and 2.2-fold increases on days 1 and 5, respectively. In HUH-7 cells, the mean number of mutations detected in FAV-exposed samples was significantly higher on D1 but not D4. These findings show the genomic stability of ZIKV varies between cell lines.

Analysis of the distribution of viral mutations in the absence of FAV in infected HeLa, SK-N-MC, and HUH-7 cells demonstrated that transition mutations were more commonly detected in all three cell lines. However, Ts/Tv ratios were markedly higher in viral samples isolated from infected HeLa cells, regardless of FAV exposure (Table 2). In the control arm, Ts/Tv ratios ranged from 3 to 4.25. These values are substantially higher than what was observed in the SK-N-MC (1.7 to 2.7) and HUH-7 (1.2 to 1.4) control samples (Table 2), highlighting the influence of cell line on the types of mutations acquired via viral replication. Ts/Tv ratios were similar between control and FAV-exposed arms in HeLa cells, but the addition of FAV tended to cause a slight increase in Ts/Tv ratios over time in SK-N-MC and HUH-7 cells, although these increases were not statistically significant with the exception of one time point (day 4 HUH-7 cells) (Table 2). Analysis of the distribution of transition mutations in HeLa cells showed that after 5 days of treatment, C→U and G→A substitutions were the most frequent transition mutations detected both in the absence of the drug and at all FAV exposures (Table 3). In SK-N-MC cells, a slightly different pattern was observed where G→A mutations were increased with the presence of FAV, C→U and A→G mutations remained similar between FAV-exposed and control arms, and U→C mutations were significantly decreased under FAV selection pressure (Table 4). For HUH-7 cells, C→U, U→C, and G→A transitions were all increased in the presence of FAV (Table 5). The increase in G→A mutations was statistically significant in the face of drug pressure relative to the control.

### 3.2. Studies to Detect the Presence of Defective Viral Particles

**Figure 1:**
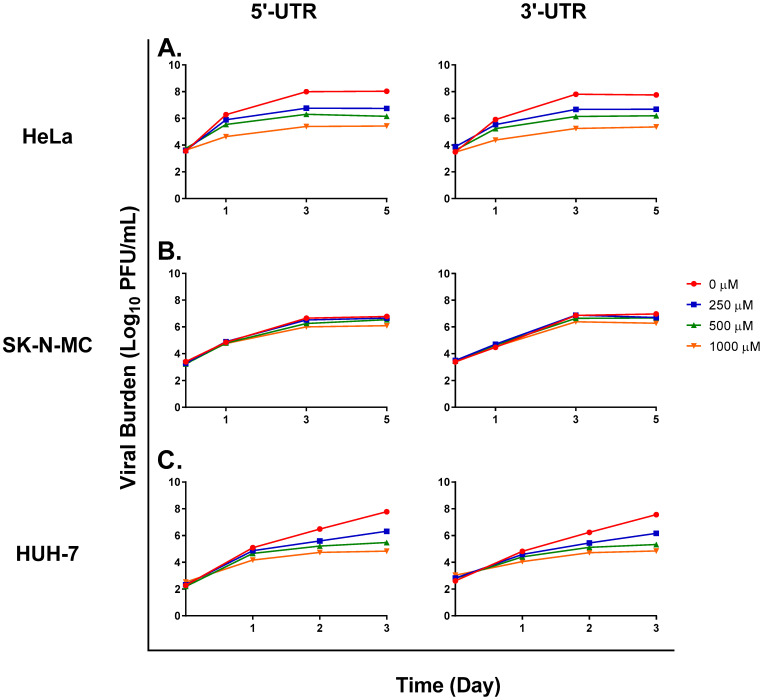
Levels of viral RNA corresponding to the 5′-untranslated region (UTR) and 3′-UTR of the ZIKV genome in HeLa, SK-N-MC, and HUH-7 cells. Viral RNA was extracted from viral supernatant samples, and amount of viral RNA in (**A**) HeLa, (**B**) SK-N-MC, and (**C**) HUH-7 cells was quantified via quantitative reverse transcription PCR. Viral burden in each treatment arm was predicted based on the amount of viral RNA present in each sample.

We sought to determine whether the presence of defective viral particles could be detected via qRT-PCR. Primers specific to the 5′ and 3′ ends of the ZIKV genome were used to detect differences in the amount of viral RNA corresponding to these regions of the ZIKV genome. If viral RNA synthesis was prematurely terminated, we reasoned that more 5′ ends of RNA would be present relative to 3′ ends since viral RNA is synthesized in a 5′-to-3′ fashion. Levels of viral RNA corresponding to the two ends of the genome were comparable in the no-treatment control arm of studies carried out using HeLa (Figure 1A), SK-N-MC (Figure 1B), and HUH-7 (Figure 1C) cells. Comparison of RNA levels in FAV-exposed treatment arms showed that the amounts of RNA corresponding to the 5′ and 3′ ends were nearly equivalent at all FAV concentrations in all three cell lines (Figure 1). These results indicate that defective particles, if present, retain their 5′ and 3′ ends, and suggest that genomic deletions may be internal rather than at the ends of the genome.

Next, we sought to detect the presence of defective particles based on their interfering activity on viral replication. We developed a competition assay where the virus that was propagated in the absence of FAV was combined with increasing ratios of virus propagated under FAV exposure. We hypothesized that increased competition from defective particles with the untreated virus would drive infectious titers down as the ratio of FAV-exposed virus increased.

**Figure 2:**
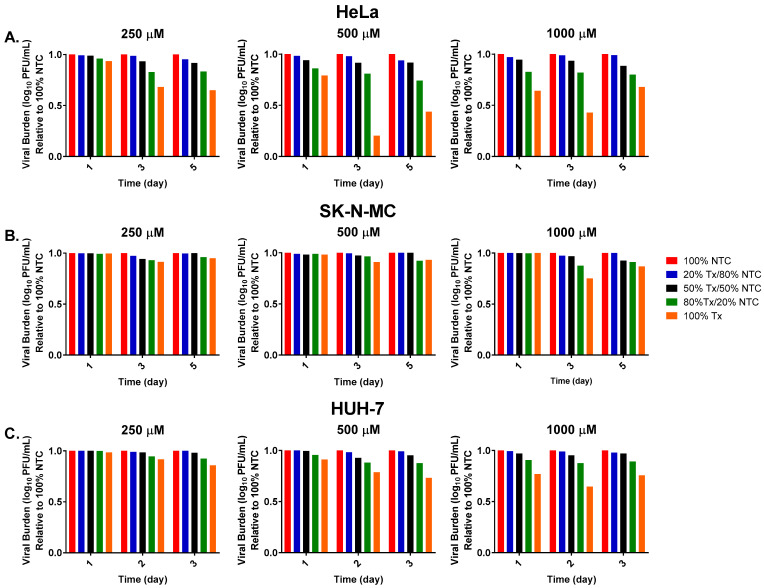
Defective particle competition assay in HeLa cells, SK-N-MC, and HUH-7 cells. ZIKV was propagated in (**A**) HeLa, (**B**) SK-N-MC, or (**C**) HUH-7 cells either in the absence or presence of FAV. No-treatment control (NTC) virus was combined with increasing ratios of FAV-exposed (Tx) virus, and infectious viral burden was quantified for all viral mixtures. Infectious titers in viral mixtures were normalized relative to viral burden in 100% NTC virus sample.

Results from a study conducted in HeLa cells showed that, as expected, infectious titers were highest in samples composed of 100% no-treatment control (NTC) virus and lowest in samples containing 100% FAV-exposed (Tx) virus since these samples are believed to contain the highest amount of defective viral particles (Figure 2A). Mixtures of NTC and Tx virus showed a very clear decrease in infectious viral burden as the ratio of Tx virus increased. This effect was observed at all evaluated drug concentrations (250, 500, and 1000 μM) and time points. The observed effect became more pronounced the longer virus was propagated in the presence of FAV. Following one day of exposure to 250 μM FAV, the viral mixture made up of 50% Tx and 50% NTC virus produced a decline in infectious titers relative to the 100% NTC of 1.2%, while the same dilution caused a drop in viral burden of nearly 8% after 5 days of exposure (Figure 2A). Declines in infectious titers were also more appreciable at higher FAV exposures. On day 1, the 80% Tx/20% NTC sample caused a drop in infectious titers of nearly 4% at 250 μM FAV, while these same dilutions reduced titers by 14% and 17% at 500 μM and 1000 μM FAV, respectively (Figure 2A).

Increasing ratios of Tx virus also reduced titers of viral mixtures in studies conducted with SK-N-MC (Figure 2B) and HUH-7 cells (Figure 2C). These findings suggest that FAV treatment also promotes the generation of defective viral particles in ZIKV-infected SK-N-MC and HUH-7 cells but not to the same extent as in HeLa cells. Studies using SK-N-MC cells showed that viral burden in mixtures of NTC and Tx virus was comparable to that in the control arm at all FAV exposures following one day of drug treatment, but the addition of Tx virus to NTC virus caused slight reductions in levels of infectious virus at the remaining time points. For example, the viral mixture composed of 80% Tx virus and 20% NTC virus in the 500 μM FAV treatment arm yielded reductions in infectious titers of approximately 4% and 8% on days 3 and 5 (Figure 2B). In HUH-7 cells, 3-day treatment with 500 μM FAV caused titers in 100% Tx samples to be 27% lower than the control virus (Figure 2C), while this same drug exposure yielded 7% and 60% drops in viral burden relative to no-treatment control samples in the virus propagated in SK-N-MC and HeLa cells, respectively. The results from these in vitro studies lend further support to our hypothesis that FAV activity against ZIKV is mediated, in part, through the generation of non-infectious defective viral particles.

**Figure 3:**
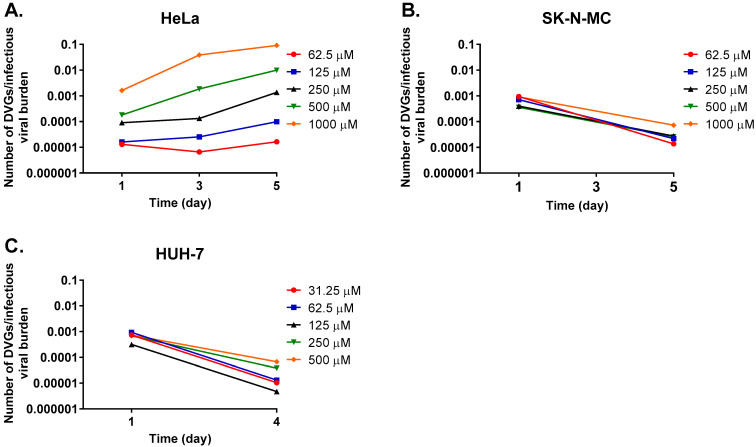
Prevalence of defective viral particles in populations of FAV-exposed ZIKV propagated in HeLa, SK-N-MC, and HUH-7 cells. The ratio of defective viral genomes to infectious viral burden was determined for ZIKV samples that were exposed to FAV and propagated in (**A**) HeLa, (**B**) SK-N-MC, and (**C**) HUH-7 cells.

To further evaluate our hypothesis that FAV’s antiviral effect is mediated through an accumulation of defective viral particles, genome sequencing data were analyzed with the program DI-tector [44], a bioinformatics tool that was developed to identify defective viral genomes (DVGs) from sequencing data. DI-tector analysis identified genome sequences corresponding to DVGs at all evaluated FAV concentrations and time points in viral supernatants collected from infected HeLa, SK-N-MC, and HUH-7 cells. In order to determine how prevalent defective viral particles are within the viral population in each sample, the ratio of the total number of defective genomes to infectious viral burden was calculated over time and at different FAV exposures. Studies performed in HeLa cells revealed a clear dose-dependent increase in the ratio of defective genomes to infectious virus (Figure 3A). On day 1, treatment with FAV at concentrations of 62.5 and 1000 μM resulted in ratios of 1.3 × 10^−5^, and 1.6 × 10^−3^, respectively. Ratios also became progressively larger throughout the course of FAV therapy, especially at FAV concentrations ≥ 250 μM; for example, the 1000 μM FAV treatment arm started at a ratio of defective particle to infectious virus of 1.6 × 10^−3^ on day 1, and the ratio then increased to 3.9 × 10^−2^ on day 3 and finally peaked at 0.1 by day 5 of FAV therapy. These results suggest that FAV treatment does in fact lead to the establishment of a growing sub-population of non-infectious virus in HeLa cells and that levels of non-infectious virus rise at increasing FAV exposures and over time. The dose-dependent increase in the ratio of defective viral genomes to infectious virus that was observed in the virus released from HeLa cells was not observed in viral samples collected from infected SK-N-MC (Figure 3B) and HUH-7 (Figure 3C) cells. Unlike results in HeLa cells, the ratio of DVGs also did not increase over time in these cell lines; instead, decreases in the ratio of DVGs to infectious virus were calculated. For example, results from a study conducted with SK-N-MC cells showed that following exposure to 1000 μM FAV, the ratio of DVGs to infectious virus decreased from 9.1 × 10^−4^ on day 1 to 7.2 × 10^−5^ on day 5 (Figure 3B). Similar declines in ratios were observed at all evaluated FAV concentrations in the virus released from HUH-7 cells (Figure 3C).

## 4. Discussion

In a companion paper [21], we investigated FAV’s antiviral potential against ZIKV in HeLa, SK-N-MC, and HUH-7 cells, three cell lines of human origin. Although our results showed that FAV curbed viral replication in all three cell lines, the antiviral effect was most pronounced in HeLa cells; our prior findings suggested that FAV’s enhanced antiviral effect occurred as a consequence of the marked declines in viral specific infectivity achieved by antiviral therapy in this cell line [21]. This observation led us to hypothesize that FAV inhibited ZIKV replication through lethal mutagenesis and that incorporation of FAV-RTP into nascent RNA strands led to the formation of mutated genomes that were packaged into non-infectious defective viral particles. These hypotheses were assessed through a variety of methods, the results of which showed that exposure to FAV increased mutation frequency as well as promoted production of defective viral particles in all three cell lines, albeit to varying degrees. Overall, the results from these studies strongly suggest that FAV inhibits ZIKV through a mechanism of action known as the lethal defection model of mutagenesis. Other laboratories have demonstrated that this mechanism explains the mutagenic activity of 5-fluorouracil (5-FU) against viruses including lymphocytic choriomeningitis virus (LCMV) and tobacco mosaic virus (TMV) [45,46,47,48,49].

One interesting finding from our investigations of FAV’s mutagenic activity was that ZIKV accumulated fewer mutations in HeLa cells compared to the SK-N-MC and HUH-7 cell lines both in the absence and presence of FAV. The fact that mutational burden increased by 2–3-fold in the absence of the drug in the virus released from SK-N-MC and HUH-7 cells but remained relatively unchanged in the virus isolated from infected HeLa cells was surprising since ZIKV replication is very robust in this cell line (peak titers of approximately 8 log_10_ PFU/mL are achieved in HeLa cells). With more rounds of replication required to achieve such high viral titers, we anticipated that more mutations would have been acquired over time in the control arm of this cell line. These results suggest that viral genomic integrity is naturally more stable in HeLa cells. The mutational burden in the virus released from infected HeLa cells was also substantially lower than that that measured in the virus isolated from infected SK-N-MC and HUH-7 cells in the face of FAV drug pressure. We considered several hypotheses to explain this observation. First, we hypothesized that if viral genomes were naturally less subject to an accumulation of mutations during replication in HeLa cells, it is possible that viral tolerance to mutations is low in this cell line; thus, the threshold to achieve lethal mutagenesis is low in HeLa cells. If this is the case, it is plausible that only a slight increase in the number of mutations as a result of drug exposure was enough to induce mutagenesis and hinder the production of infectious virus. Another potential explanation for this observation is that since FAV causes a loss of infectious viral burden over time and defective particles are unable to effectively replicate themselves, it becomes less possible to achieve the same level of mutagenesis in HeLa cells as in the other cell lines due to declines in the viral population in drug-exposed experimental arms. These findings highlight the importance of the careful selection of cell lines for antiviral evaluations, as host cell factors likely have a strong influence on the degree of antiviral activity and mechanism of action due to the intrinsic differences that exist between cell lines.

Prior research on defective viral particles has indicated that as levels of defective viral particles increase within a viral population, competition between replication-competent and defective viruses limits the amount of infectious virus that can be produced and released from host cells, thus leading to declines in viral infectivity and possible viral extinction [32,45]. Since viral inhibition through this mechanism hinges on the accumulation of defective viral particles, it stands to reason that we observed the greatest antiviral effect in HeLa cells as ratios of DVGs to infectious virus indicated that treatment caused defective viral particles to become more prevalent in the viral population both at increasing FAV concentrations and at increasing exposure times. It is important to note that defective particles were also present in samples of the virus released from infected SK-N-MC and HUH-7 cells. However, when levels of defective genomes were compared to infectious virus, infectious virus was much more prevalent in viral populations released from these cell lines. This explains why the drug effect on viral infectivity was less pronounced in these two cell lines.

Since FAV is administered as a prodrug that must be taken up by the cell and converted into an active triphosphate metabolite by host cell kinases, it is plausible that tissue-specific variability in the ability to accumulate FAV inside the cell and in activation of FAV into FAV-RTP may explain observed differences in susceptibility to the drug effect among tissues. Prior work performed in the lab [19] as well as by others [50] has established that intracellular levels of FAV and FAV-RTP vary between cell lines, thus lending support to this hypothesis. Because FAV exposure elicited an antiviral effect in HeLa, SK-N-MC, and HUH-7 cells, we can conclude that FAV was able to both enter and undergo phosphorylation in evaluated cell types. Findings from our prior work with FAV against chikungunya virus (CHIKV) in a range of human-derived cell lines [19] demonstrated that the cell type that was most sensitive to FAV’s anti-CHIKV effect had the highest measured levels of intracellular FAV parent drug and active metabolite. Therefore, we postulate that differences in the degree of intracellular accumulation of FAV and FAV-RTP exist between tissue/cell types and that these differences contribute to the disparities in drug effect observed here. If this is the case, it is likely that intracellular concentrations of FAV and FAV-RTP are highest in HeLa cells since FAV’s antiviral and defective particle effect were most pronounced in this cell line. In this scenario, abundant quantities of intracellular FAV would facilitate accumulation of high levels of FAV-RTP since a large pool of substrate would be available inside the cell for conversion into the triphosphate metabolite. Others have suggested that the concentration of analogue relative to natural nucleotide plays a role in determining the effectiveness of a nucleoside analogue in a given tissue [51]. If high concentrations of FAV-RTP were achieved in HeLa cells, it is possible that intracellular concentrations of drug were high enough in this cell line for FAV-RTP to outcompete the natural purines ATP and GTP for incorporation into nascent RNA strands, thereby leading to an increased likelihood of analogue incorporation and increased production of mutated viral genomes that could be packaged as defective viral particles. Future studies will focus on quantifying FAV-RTP levels in relation to the natural purines in each cell line in order to determine whether differences in the ratios of analogue to natural substrate explain disparities in FAV’s antiviral effect.

Overall, the results from this study and our companion paper [21] indicate that further investigation of FAV’s therapeutic potential against ZIKV is warranted since drug exposure was able to elicit a strong antiviral response from HeLa cells. Since ZIKV can be transmitted sexually and is known to infect and persist in tissues of the reproductive tract [52,53], results in HeLa cells showing that FAV induces a shift toward production of non-infectious virus are especially promising since they suggest that FAV treatment may be considered as a method to curb sexual transmission of ZIKV. Although its effect was moderate in comparison, FAV was also able to limit viral replication in SK-N-MC cells. In order to make FAV treatment more effective in neuronal cells, we may need to consider ways to increase drug exposure inside the cell, for example by increasing doses or increasing frequency of administration. Alternatively, the use of combination therapy with a second agent that has a distinct mechanism of action may be considered as a method to maximize viral suppression in this cell type.

One limitation associated with this study is that static drug concentrations were utilized; this scenario does not accurately represent the fluctuating levels of the drug found in humans following FAV administration. In future studies, we will use dynamic in vitro models to simulate clinical FAV regimens in order to determine whether human exposures elicit a similar mutagenic response compared to static drug concentrations. We will also use these systems to characterize FAV phosphorylation kinetics under dynamic drug concentrations to determine whether there are differences in FAV’s ability to be taken up and metabolized by these host cells.

The results of these studies revealed that the antiviral mechanism of action responsible for FAV’s antiviral activity against ZIKV is likely activity as a mutagen, although this does not exclude the possibility that other mechanisms of action also contribute to FAV’s effect. Our findings also highlight the host cell’s impact on the activation and antiviral activity of nucleoside analogues and suggest that when considering potential treatment options against ZIKV and other pantropic viruses, it is important to understand differences in antiviral activity between tissue types since this understanding can be used to optimize treatment regimens.

## Figures and Tables

**Table 1:** Number of mutations in the absence or presence of FAV in virus released from infected HeLa, SK-N-MC, and HUH-7 cells.

	HeLa			SK-N-MC			HUH-7	
	Mutations (n)			Mutations (n)			Mutations (n)	
FAV Exposure (μM)	D1	D3	D5	D1	D5	FAV Exposure (μM)	D1	D4
0	21	22	25	40	78	0	30	92
62.5	22	38	36	48	194	31.25	74	82
125	21	34	36	48	131	62.5	59	48
250	21	29	35	73	175	125	113	111
500	24	37	27	76	182	250	130	125
1000	27	22	31	66	176	500	125	130
Mean Drug (95% CI)	23 (19.8–26.2)	**32** **(23.8–40.2)**	**33** **(28.1–37.9)**	**62.2** **(45.5–78.9)**	**171.6** **(141.9–201.3)**	Mean Drug (95% CI)	**100.2** **(60.7–139.7)**	99.2 (56.8–141.6)

**Table 2:** Transition/transversion (Ts/Tv) ratios for ZIKV propagated in the absence or presence of FAV in HeLa, SK-N-MC, and HUH-7 cells.

	HeLa			SK-N-MC			HUH-7	
	Ts/Tv Ratio			Ts/Tv Ratio			Ts/Tv Ratio	
FAV Exposure (μM)	D1	D3	D5	D1	D5	FAV Exposure (μM)	D1	D4
0	4.25	3.4	3	1.7	2.7	0	1.4	1.2
62.5	7	2	3	2.17	2.64	31.25	2.2	2.5
125	3	3.8	3.6	1.43	2.9	62.5	1.7	4.1
250	3.2	4.6	5.5	2	2.7	125	3.2	3.5
500	3.4	3.6	3.75	1.7	2.2	250	2.1	4.3
1000	3.5	3.2	2.83	2.1	3.3	500	1.5	5.4
Mean Drug (95% CI)	4 (1.9–6.1)	3.4 (2.3–4.6)	3.7 (2.4–5)	1.9 (1.5–2.3)	2.8 (2.3–3.2)	Mean Drug (95% CI)	2.1 (1.3–2.9)	**4** **(2.6–5.3)**

**Table 3:** Distribution of transition mutations following 5 days of FAV treatment in HeLa cells.

	Number of Mutations			
FAV Exposure (μM)	C→U	U→C	A→G	G→A
0	8	2	3	4
62.5	7	2	3	5
125	7	2	3	6
250	8	2	4	8
500	7	1	3	4
1000	8	1	3	5
Mean Drug (95% CI)	7.4 (6.7–8.1)	1.6 (0.9–2.3)	3.2 (2.6–3.8)	5.6 (3.7–7.5)

**Table 4:** Distribution of transition mutations after 5 days of FAV treatment in SK-N-MC cells.

	Number of Mutations			
FAV Exposure (μM)	C→U	U→C	A→G	G→A
0	9	3	3	4
62.5	11	2	4	5
125	9	1	3	8
250	9	3	2	5
500	6	2	7	6
1000	8	1	3	8
Mean Drug (95% CI)	8.6 (6.3–10.9)	**1.8 (0.8–2.8)**	3.8 (1.4–6.2)	**6.4 (4.5–8.3)**

**Table 5:** Distribution of transition mutations after 4 days of FAV treatment in HUH-7 cells.

	Number of Mutations			
FAV Exposure (μM)	C→U	U→C	A→G	G→A
0	8	2	3	5
31.25	8	1	3	6
62.5	11	7	4	12
125	7	4	1	8
250	12	3	4	10
500	12	6	5	14
Mean Drug (95% CI)	10 (7.1–12.9)	4.2 (1.2–7.2)	3.4 (1.5–5.3)	**10 (6.1–13.9)**

## Data Availability

The data presented in this study are available on request from the corresponding author.
